# Rationale and design of a randomized trial to test the safety and non-inferiority of canagliflozin in patients with diabetes with chronic heart failure: the CANDLE trial

**DOI:** 10.1186/s12933-016-0381-x

**Published:** 2016-04-04

**Authors:** Atsushi Tanaka, Teruo Inoue, Masafumi Kitakaze, Jun-ichi Oyama, Masataka Sata, Isao Taguchi, Wataru Shimizu, Hirotaka Watada, Hirofumi Tomiyama, Junya Ako, Yasushi Sakata, Toshihisa Anzai, Masaaki Uematsu, Makoto Suzuki, Kazuo Eguchi, Akira Yamashina, Yoshihiko Saito, Yasunori Sato, Shinichiro Ueda, Toyoaki Murohara, Koichi Node

**Affiliations:** Department of Cardiovascular Medicine, Saga University, Saga, Japan; Department of Cardiovascular Medicine, Dokkyo Medical University School of Medicine, Mibu, Japan; Department of Clinical Medicine and Development, National Cerebral and Cardiovascular Center, Suita, Japan; Department of Advanced Cardiology, Saga University, Saga, Japan; Department of Cardiovascular Medicine, Institute of Biomedical Sciences, Tokushima University Graduate School, Tokushima, Japan; Department of Cardiology, Koshigaya Hospital, Dokkyo Medical University, Koshigaya, Japan; Department of Cardiovascular Medicine, Nippon Medical School, Tokyo, Japan; Department of Metabolism & Endocrinology, Juntendo University Graduate School of Medicine, Tokyo, Japan; Department of Cardiology, Tokyo Medical University, Tokyo, Japan; Department of Cardiovascular Medicine, Kitasato University School of Medicine, Sagamihara, Japan; Department of Cardiovascular Medicine, Osaka University Graduate School of Medicine, Suita, Japan; Department of Cardiovascular Medicine, National Cerebral and Cardiovascular Center, Suita, Japan; Kansai Rosai Hospital Cardiovascular Center, Amagasaki, Japan; Cardiology Department, Kameda Medical Center, Kamogawa, Japan; Division of Cardiovascular Medicine, Department of Medicine, Jichi Medical University, Shimotsuke, Japan; First Department of Internal Medicine, Nara Medical University, Kashihara, Japan; The Clinical Research Center, Graduate School of Medicine, Chiba University, Chiba, Japan; Department of Clinical Pharmacology & Therapeutics, University of the Ryukyus School of Medicine, Okinawa, Japan; Department of Cardiology, Nagoya University Graduate School of Medicine, Nagoya, Japan

**Keywords:** Canagliflozin, Chronic heart failure, Glimepiride, Non-inferiority, NT-proBNP, Safety, SGLT2 inhibitor, Type 2 diabetes mellitus

## Abstract

**Background:**

Because type 2 diabetes mellitus is associated strongly with an increased risk of cardiovascular diseases, the number of patients with diabetes with chronic heart failure is increasing steadily. However, clinical evidence of therapeutic strategies in such patients is still lacking. A recent randomized, placebo-controlled trial in patients with type 2 diabetes with high cardiovascular risk demonstrated that the SGLT2 inhibitor, empagliflozin, reduced the incidence of hospitalization for heart failure. Because SGLT2 inhibitors cause a reduction in body weight and blood pressure in addition to improving glycemic control, they have the potential to exert beneficial effects on the clinical pathophysiology of heart failure. The aim of the ongoing CANDLE trial is to test the safety and non-inferiority of canagliflozin, another SGLT2 inhibitor, compared with glimepiride, a sulfonylurea agent, in patients with type 2 diabetes mellitus and chronic heart failure.

**Methods:**

A total of 250 patients with type 2 diabetes who are drug-naïve or taking any anti-diabetic agents and suffering from chronic heart failure with a New York Heart Association classification I to III will be randomized centrally into either canagliflozin or glimepiride groups (1: 1) using the dynamic allocation method stratified by age (<65, ≥65 year), HbA1c level (<6.5, ≥6.5 %), and left ventricular ejection fraction (<40, ≥40 %). After randomization, all the participants will be given the add-on study drug for 24 weeks in addition to their background therapy. The primary endpoint is the percentage change from baseline in NT-proBNP after 24 weeks of treatment. The key secondary endpoints after 24 weeks of treatment are the change from baseline in glycemic control, blood pressure, body weight, lipid profile, quality of life score related to heart failure, and cardiac and renal function.

**Discussion:**

The CANDLE trial is the first to assess the safety and non-inferiority of canagliflozin in comparison with glimepiride in patients with type 2 diabetes with chronic heart failure. This trial has the potential to evaluate the clinical safety and efficacy of canagliflozin on heart failure.

*Trial registration* Unique trial Number, UMIN000017669

**Electronic supplementary material:**

The online version of this article (doi:10.1186/s12933-016-0381-x) contains supplementary material, which is available to authorized users.

## Background

Co-morbidities associated with type 2 diabetes mellitus (T2DM) are increasing steadily in patients with chronic heart failure (CHF). Their prevalence was reported to be 30 % in the JCARE-CARD trial and 23 % in the CHART-2 trial [1, 2]. Complications of T2DM aggravate clinical outcomes, such as higher mortality, morbidity, and re-hospitalization rate for worsening heart failure in patients with CHF. This indicates that T2DM is a relatively strong risk factor for CHF [3, 4]. Although glycemic control is recognized as essential, a U-curve phenomenon is found between HbA1c levels and mortality in CHF patients with T2DM [5]. The treatment guidelines for CHF of the Japanese Circulation Society are similar to those of the American Heart Association and European Society of Cardiology in that the established evidence on the recommended therapeutic strategies in T2DM are not provided [6]. In addition, the Japan Diabetes Society has also not described specific treatment options for diabetes in patients with CHF, although lowering HbA1c levels to <7.0 % is recommended in order to suppress the development and progression of microvascular complications [7].

**Fig. 1:**
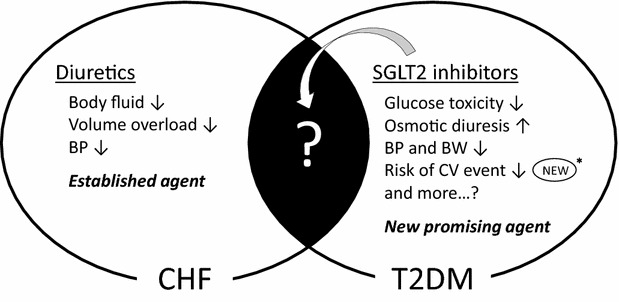
Trial concept. Conventional diuretics are well established agents for CHF. On the other hand, SGLT2 inhibitors are new anti-diabetic agents with multiple favorable effects such as improving glycemic and metabolic parameters. In addition, empagliflozin has recently been reported to reduce the risk of CV events *asterisk* [13]. However, the beneficial effects of SGLT2 inhibitors in T2DM patients with CHF have yet to be established. *BP* blood pressure, *BW* body weight, *CHF* chronic heart failure, *CV* cardiovascular, *SGLT2* sodium glucose cotransporter 2, *T2DM* type 2 diabetes mellitus

Sodium glucose co-transporter 2 (SGLT2) inhibitors are a novel class of anti-diabetic agents that have a blood glucose-lowering effect by increasing urinary glucose excretion [8, 9]. In addition to this glucose-lowering effect, previous studies have demonstrated that SGLT2 inhibitors decrease blood pressure (BP) due to their osmotic diuretic action, and also induce metabolic ameliorations, including a reduction of visceral fat and body weight (BW) [10–12]. The diuretic action of SGLT2 inhibitors may also have advantages on heart failure. Recently, it has been reported that administration of empagliflozin, a SGLT2 inhibitor, as an add-on to conventional anti-diabetic therapy, significantly reduced cardiovascular (CV) adverse outcomes in T2DM patients with higher CV risk [13]. Given their pleiotropic pharmacological actions, SGLT2 inhibitors appear to contribute to amelioration of the clinical course of T2DM patients with CHF by reducing both blood glucose and excess body fluid. Canagliflozin is another SGLT2 inhibitor and the first-in-class drug launched in the United States. In phase III clinical trials, canagliflozin was reported to cause favorable long-term glycemic and metabolic improvement and was well tolerated as either mono-therapy or in combination with other anti-diabetic agents [14–16]. Another randomized placebo-controlled trial designed to assess the longer-term effects of canagliflozin on clinical outcomes in T2DM patients is currently being carried out [17]. To enhance the favorable class effect of SGLT2 inhibitors on cardiovascular events, it is important to refer to additional trials on other SGLT2 inhibitors. However, T2DM patients with severe CHF with a New York Heart Association (NYHA) functional classification of III and IV were excluded from these trials, because the use of SGLT2 inhibitors is not clinically recommended in these patients. As a consequence, the clinical efficacy and safety of SGLT2 inhibitors in T2DM patients with CHF remains to be elucidated (Fig. 1).

The CANDLE trial was designed to test the safety and non-inferiority of canagliflozin, compared with glimepiride, using N-terminal pro-brain natriuretic peptide (NT-proBNP) as the index of the therapeutic effects in T2DM patients with CHF. This trial has the potential to provide novel clinical evidence regarding the safety and efficacy of canagliflozin as anti-diabetic therapy in T2DM patients with CHF.

## Methods

### Study overview and design

The CANDLE trial is an ongoing, multicenter, prospective, randomized, open-label, blinded-endpoint investigator-initiated clinical trial. This study tests the hypothesis that additional administration of canagliflozin to standard therapy does not worsen the pathological state of CHF, compared to the conventional anti-diabetic agent, glimepiride. Twenty-four weeks after recruitment and randomization into either canagliflozin or glimepiride groups, the safety and non-inferiority of canagliflozin for T2DM patients with CHF will be evaluated using NT-proBNP as a biomarker of heart failure.

Prior to initiation, the study protocol needs to be approved by the local institutional review boards and independent ethics committees at every site. The trial will be conducted in full compliance with the Declaration of Helsinki and according to the Ethical Guidelines for Medical and Health Research Involving Human Subjects established by the Ministry of Health, Labour, and Welfare and Ministry of Education, Culture, Sports, Science, and Technology.

### Trial population and recruitment

We aim to recruit a total of 250 participants across approximately 35 sites in Japan. Recruitment for this trial began in October 2015 and will be completed by December 2017. Eligible patients in the trial are T2DM patients with CHF (aged ≥20 years) who comply with all the enrollment criteria. The detailed inclusion and exclusion criteria are listed in Table 1. Briefly these criteria include: (1) Patients with appropriately diagnosed T2DM, who are drug-naïve or taking any anti-diabetic agent; (2) T2DM clinically requiring a start or change of an anti-diabetic agent; (3) complicating NYHA functional classification I to III CHF, but not IV, with maintenance of the clinical condition and unchanged medical treatment for CHF, including angiotensin-converting enzyme inhibitors, angiotensin II receptor blockers, beta-blockers, and diuretic agents for 4 weeks prior to screening. In the trial, CHF is classified comprehensively by skilled cardiologists using several findings, including clinical symptoms (the Framingham criteria for congestive heart failure and NYHA functional classification), history of hospitalization for heart failure, and clinical tests such as echocardiography and biomarkers. However, there is no limitation on the NT-proBNP level at enrollment. Prior to eligibility screening, every participant is required to receive an adequate explanation based of the trial plan, with written informed consent then being obtained from each patient.

**Table 1:** Detailed inclusion and exclusion criteria

Inclusion	Exclusion
Adults (aged ≥20 years)	Type 1 diabetes mellitus
T2DM patients who need to start or who are possibly changing or adding an anti-diabetic agent	History of diabetic ketoacidosis, diabetic coma, or hypoglycemic attack ≤6 months prior to informed consent
CHF (NYHA functional classification I to III)	Severe renal dysfunction (eGFR <45 ml/min/1.73 m^2^) or patients receiving dialysis
Without change in NYHA functional classification or drugs for heart failure 4 weeks prior to eligibility	Severe liver dysfunction (at least threefold higher AST or ALT more than the upper limit of the facilities reference value
The patient provided written informed consent to participate in the study	CHF (NYHA functional classification IV)
	Patients with pituitary or adrenal dysfunction
	Patients with malnutrition, starvation, irregular eating pattern, lack of dietary intake, or debilitation
	Patients with excess alcohol intake
	Patients in perioperative period around trial screening
	Patients with severe infection or trauma at trial screening
	Patients with a gastrointestinal disorder, such as diarrhea or vomiting
	Patients with low body weight (BMI **<**18.5 kg/m^2^)
	History of coronary artery disease, coronary vascularization, open-heart surgery, stroke, or transient ischemic attack ≤3 months prior to eligibility
	Patients with a malignancy
	History of hypersensitivity to ingredients of SGLT2 inhibitors or sulfonylureas
	Pregnant or suspected pregnancy in females
	Lactating female
	Considered inappropriate for the study by investigators due to other reasons

*ALT* alanine aminotransferase, *AST* aspartate transaminase, *BMI* body mass index, *CHF* chronic heart failure, *eGFR* estimated glomerular filtration rate, *NYHA* New York Heart Association, *SGLT2* sodium glucose cotransporter 2, *T2DM* type 2 diabetes mellitus

### Trial outline and follow-up

**Fig. 2:**
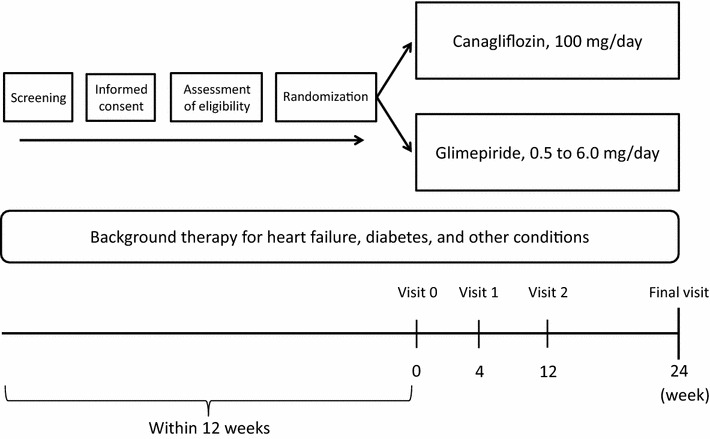
Trial outline

After informed consent has been provided and the eligibility assessment completed, all eligible participants will be randomized and assigned into either the canagliflozin or glimepiride groups. Post-randomized follow-up visits are scheduled at 4, 12, and 24 weeks (Fig. 2). All participants will see their usual-care physicians at each visit to receive usual-care and individualized appropriate treatment according to their background disease, T2DM, and CHF, in addition to administration of the study drug.

### Randomization and treatment

Eligible patients with the appropriately signed informed consent will be randomized to either the canagliflozin or glimepiride groups at a ratio of 1:1 using the minimization method with biased coin assignment balancing for age (<65, ≥65 year), HbA1c level (<6.5, ≥6.5 %), and left ventricular ejection fraction (LVEF; <40, ≥40 %) at the time of screening [18, 19]. The trial is an open-label design, although the assessor(s) will be blinded to treatment randomization. The assessors will also be blinded to the primary end point, NT-proBNP level, by measurement at a central laboratory.

All participants receive the study drug for 24 weeks. Patients who are assigned to the canagliflozin group receive canagliflozin 100 mg once daily. The EMPA-REG OUTCOME trial reported small dose-dependent effects of empagliflozin (10 mg vs. 25 mg/day) on metabolic parameters. However, the two dose groups had similar hazard ratios for cardiovascular outcomes [13]. Some studies on canagliflozin showed that 100 mg/day had sufficient effects on metabolic parameters, while 300 mg/day was slightly more effective [14, 15, 20]. Importantly, only 100 mg/day is approved in Japan, and therefore the dose of canagliflozin is fixed at 100 mg/day in the canagliflozin group. Because of the possible risk of excess diuresis and subsequent dehydration by combined use of canagliflozin and diuretics, the dose of the diuretics may need to be reduced when necessary. Patients assigned to the glimepiride group are started at a dose of 0.5 or 1.0 mg once or twice daily. According to the individual’s glycemic control, the dose of glimepiride can be increased to 6.0 mg/day. For background therapy in both groups, participants who have been receiving any SGLT2 inhibitor other than canagliflozin need to discontinue it at the start of the washout period, for 4 weeks, prior to receiving the study drug, canagliflozin or glimepiride. While, participants who have been taking a sulfonylurea need to stop taking it in the canagliflozin group and need to change to glimepiride (if needed) in the glimepiride group at the initiation of the study drug (Additional file 1). Because of the possible risk of excess diuresis and subsequent dehydration by combined use of canagliflozin and diuretics, the dose of the diuretics may need to be reduced when necessary.

Although a specific numerical goal of HbA1c level is not set in this trial, all participants need to be treated to achieve a personalized goal recommended by the guidelines (details in Additional file 2) [7]. Drugs that must not be used in combination with the study drugs are listed in Table 2. Within the appropriate range of the therapeutic goal, the participant’s background treatment will be, in principle and if possible, unchanged during the trial interval. If participants cannot achieve their glycemic goal, co-administration of anti-diabetic agents other than SGLT2 inhibitors and sulfonylureas or increased dosages of the anti-diabetic agents other than the target agents in both groups may be initiated, with caution being taken to prevent the development of hypoglycemia. After the trial is completed, all participants can continue any anti-diabetic treatment in accordance with their individual condition.

**Table 2:** Prohibited concomitant drugs

Canagliflozin group
SGLT2 inhibitors other than canagliflozin
Sulfonylureas
Pioglitazone
Glimepiride group
Sulfonylureas other than glimepiride
SGLT2 inhibitors
Pioglitazone

*SGLT2* sodium glucose cotransporter 2

### Measurements

Baseline characteristics, including smoking habit, NYHA functional classification, etiology of CHF, hypertension, dyslipidemia, and other complications will be screened prior to randomization. The medication status of the study agents, and the participant’s background treatment, BW, BP, and heart rate will be also measured at each visit. A chest X-ray is taken at baseline and at the final visit. An echocardiogram is performed at screening, baseline, and the final visit to measure LVEF using the modified Simpson method and E/e’. The echocardiograms will be evaluated by technicians at each local site. Blood and urine tests will be checked at each visit (details listed in Additional file 3). Specific biomarkers, including NT-proBNP, high-sensitivity C reactive protein (hsCRP), serum cystatin C, pentraxin 3 (PTX3) (SRL, Inc. Tokyo, Japan), high-sensitivity troponin I (Abbot Japan, Tokyo, Japan), receptor for advanced glycation end products (RAGE), angiopoietin-like protein 2 (ANGPTL2), interleukin 8 (IL-8), and periostin (Saga University) will be measured at a central laboratory at baseline and the final visit. CHF-related quality of life is evaluated by scaled responses to the Minnesota Living with Heart Failure (MLHF) questionnaire at baseline and the final visit. The occurrence of CV events, including all-cause death, non-fatal myocardial infarction, non-fatal stroke, hospitalization for heart failure, and additional medications or dosage increases of drugs due to aggravation of heart failure will be evaluated.

### Safety

Based on the intention-to-treat entire population, safety will be checked by recording the following adverse effects throughout the duration of the study: hypoglycemia, genital infections, urinary tract infections, excess osmotic diuresis signs (increased urinary frequency, mouth dryness, or polyuria), and hypovolemic symptoms. A previous study has reported an increased prevalence of hypoglycemia with sulfonylureas than with canagliflozin [14]. We therefore need to pay careful attention to the development of hypoglycemia, especially in patients assigned to the glimepiride group, despite this drug being initiated at a low dose in the study. If the investigators confirm these adverse effects, the grade of severity, treatment, outcomes, and relationship to the study drug will be assessed. The criteria for withdrawal from the trial are listed in Table 3. In addition, the incident of withdrawal from the study will be reported promptly to the Data and Safety Monitoring Board (DSMB) by the trial organizer.

**Table 3:** Discontinuance criteria

Severe hypoglycemia that needs some support by family members or medical care at medical settings
Seriously poor glycemic control such as ≥HbA1c 10.0 %
Diabetic ketoacidosis
Dehydration
Considered inappropriate to continue the study by investigators due to aggravation of primary disease or complications
Considered inappropriate to continue the study by investigators due to adverse side effects of the study drug
Offer for participation declined by participants
Considered inappropriate to continue the study by investigators due to some other reason

### Trial endpoints

NT-proBNP is used widely to monitor the therapeutic efficacy of agents for heart failure in clinical trials and is established as a prognostic biomarker for heart failure [21–23]. In addition, NT-proBNP-guided treatment for heart failure has been demonstrated to improve clinical outcomes in a randomized trial [24]. Therefore, the percent change in NT-proBNP levels from baseline to 24 weeks will be used as the primary endpoint in the CANDLE trial. The secondary endpoints are the values and changes in parameters after 24 weeks of treatment (or at discontinuation), including: (1) NT-proBNP, (2) glycemic control (HbA1c, fasting plasma glucose, plasma insulin, HOMA-R and HOMA-beta), (3) clinic systolic and diastolic BP and home BP (optional), (4) BW, (5) biochemical tests (lipid profiles and uric acid), (6) quality of life (MLHF scores) (7) echocardiographic cardiac function parameters (LVEF and E/e’) and severity and change in NYHA functional classification, and (8) renal function parameters (serum creatinine level, estimated glomerular filtration rate (eGFR), urinary albumin excretion, and urine L-FABP). Other secondary endpoints include clinical outcomes such as CV events, including all-cause death, non-fatal myocardial infarction, non-fatal stroke, hospitalization for heart failure, and changes in medications related to aggravation of heart failure. Safety endpoints include adverse effects and adverse drug reactions observed during treatment with the study drugs. The exploratory endpoints are changes in specific biomarkers after 24 weeks of treatment, including high-sensitivity troponin I, hsCRP, serum cystatin C, PTX3, RAGE, ANGPTL2, IL-8, and periostin. The findings of these biomarker assays may, in part, provide mechanistic insights on the effect of canagliflozin on CV pathogenesis.

### Endpoint adjudication

A clinical event committee (CEC) blinded to treatment allocation will independently adjudicate the clinical events. The DSMB blinded to treatment allocation will also independently evaluate safety during the study period. The DSMB will also assess the necessity for revision of the trial design and the validity for continuance of trial entry, and if needed, recommend issues for the chief investigator. The members of the CEC and DSMB consist of authorized cardiologists or endocrinologists with relevant expertise.

### Statistical considerations

#### Sample size estimation

Due to the lack of data on the effect of SGLT2 inhibitors, including canagliflozin, on NT-proBNP levels, we used data from the PARAMOUNT study on CHF patients with preserved ejection fraction [25]. That study used the same primary end point to compare the efficacy and safety of LCZ696, an angiotensin receptor neprilysin inhibitor, with valsartan. On the basis of the findings of that study, we assumed an 18 % difference in NT-proBNP reduction between the two groups in our trial. In detail, estimation of the sample size for the CANDLE trial was based on the percentage change from baseline to the final NT-proBNP level, required to demonstrate non-inferiority of canagliflozin versus glimepiride, using a one-sided Student t test with a 2.5 % type I error. In this calculation we assumed that for a common standard deviation for the log-scale of the ratio of 0.80, a clinical non-inferiority margin of 1.1, and a dropout rate of 10 %, it was necessary to recruit 125 patients in each group in the full analysis set (FAS) to demonstrate non-inferiority if the true difference was 18 % and the power was at least 80 %.

#### Analysis plan

The analyses of the primary and secondary endpoints will be performed in the FAS, which includes all patients who received at least one dose of treatment during the study period and did not have any serious violation of the study protocol, and had data collected after commencement of treatment. For the baseline characteristics, the summary statistics will comprise frequencies and proportions for categorical variables, and means and standard deviations for continuous variables. The patient characteristics will be compared using Chi square tests for categorical variables, and t tests for normally distributed continuous variables or Wilcoxon rank sum tests for continuous variables with a skewed distribution.

For the primary analysis comparing treatment effects, the least square mean and its 95 % confidence interval will be estimated using analysis of covariance (ANCOVA). To test for non-inferiority of the primary endpoint, the ANCOVA will be applied adjusted for age (<65, ≥65 year), HbA1c level (<6.5, ≥6.5 %), and left ventricular ejection fraction (LVEF; <40, ≥40 %). The secondary analysis will be performed in the same manner as the primary analysis.

All comparisons are planned and all *p* values will be two sided. *P* values <0.05 will be considered statistically significant. All statistical analyses will be performed using SAS software version 9.4 (SAS Institute, Cary, NC, USA). The statistical analysis plan will be developed by the principal investigator and a biostatistician before completion of patient recruitment and database lock.

Trial organization and oversight (details in Additional file 4).

Principal investigators of the CANDLE trial are Koichi Node (Chief), Department of Cardiovascular Medicine, Saga University and Toyoaki Murohara, Department of Cardiology, Nagoya University Graduate School of Medicine. The Steering Committee will carry out planning, operating, analyzing, and presentation of the trial. The Executive Committee will supervise the trial design and operation of the study. The roles of DSMB and CEC are described in the section of Endpoint adjudication. The trial secretariat is in DOT INTERNATIONAL CO., LTD, Tokyo, Japan. Each of data management, monitoring, statistical analyses, and audit will be independently implemented on the basis of outsourcing agreement.

## Discussion

The CANDLE trial is an ongoing, multicenter, prospective, randomized, investigator-initiated clinical trial that has the aim of testing the safety and non-inferiority of canagliflozin in T2DM patients with CHF. In this trial, eligible participants will be randomized to canagliflozin or glimepiride groups and receive the study drug in addition to their background medications for 24 weeks. The primary endpoint is the percentage change from baseline in NT-prBNP level after 24 weeks of treatment. This trial has the potential to generate novel evidence regarding the clinical relevancy of canagliflozin in T2DM patients with CHF.

Accumulating evidence suggests that T2DM has a major impact on the increased risk of micro- and macrovascular complications that are associated strongly with unfavorable clinical outcomes and poor prognosis [26–29]. Importantly, comprehensive medical intervention in diabetic management is encouraged at earlier stages of disease development in order to improve patient prognosis [30–32]. Although previous studies have shown that glucose-lowering therapy is important in the clinical setting, the beneficial effects on long-term outcomes and prognosis remain controversial [33–37]. For the management of diabetic patients, current guidelines state therapeutic goals for HbA1c levels, based on the patient’s background and tolerability to treatment [7]. In addition, for safety reasons it is essential that anti-diabetic agents do not increase the risk of CV events, and at least, are not inferior to conventional therapy [38]. Nevertheless, despite the increasing number of T2DM patients complicated with CHF, in whom a higher risk of CV events is possible, there is no clinical evidence on therapeutic goals based on safety and efficacy in this population. Therefore, establishment of therapeutic guidelines for T2DM patients with CHF is required.

Of the various conventional anti-diabetic agents, thiazolidine derivatives are effective for improving insulin resistance by enhancing insulin sensitivity in peripheral tissues, but need to be administered with caution because of possible adverse effects such as worsening of edema and heart failure [39, 40]. Dipeptidyl peptitadase-4 inhibitors have been shown to have neutral effects on CV outcome in both the EXAMINE (alogliptin vs. placebo) and TECOS (sitagliptin vs. placebo) trials [41, 42]. However, the SAVOR-TIMI53 trial (saxagliptin vs. placebo) demonstrated recently a significantly higher incidence of hospitalization for heart failure in the saxagliptin group, compared to placebo [43]. Therefore, at present the clinical use of anti-diabetic agents in T2DM patients with CHF is, in part, somewhat limited. As a consequence, although these patients are considered to be at higher CV risk and require more effective treatment strategies to improve their clinical outcome, no specific treatment guidelines have been established. Metformin is recognized widely as the first line strategy for treatment of type 2 diabetes in the USA and Europe, with some study showing a preference for metformin over sulfonylureas in diabetes patients with heart failure [44, 45]. However, metformin is not necessarily a first line treatment option in Japan [7]. Although the present study excludes patients with renal dysfunction who are prohibited to receive metformin, metformin is contraindicated in Japan for patients with heart failure because of the risk of lactic acidosis. In addition, it has been reported that decreased insulin secretion capacity has a pivotal role in the development of T2DM in Japanese subjects and accordingly sulfonylureas are used more frequently than metformin in Japan [46]. To our knowledge, there is no direct evidence showing that sulfonylureas increase the risk of heart failure. A phase III trial in the USA showed sulfonylureas had similar power to decrease HbA1c levels as canagliflozin 100 mg/day [14]. We therefore selected glimepiride as the comparison agent.

Previous clinical trials in T2DM patients demonstrated that SGLT2 inhibitors are well tolerated by patients and exert favorable effects on BP and systemic metabolisms such as body fat loss, in addition to improving glycemic control [10–12]. Experimental studies using animal models have shown that SGLT2 inhibitors also reduce inflammation and oxidative stress in hepatic, renal, and cardiac tissues [47–50], and therefore presumably would have a protective effect on the CV system. These class effects would be expected to be highly advantageous for diabetic patients with increased CV risk, while the diuretic effects of SGLT2 inhibitors may also be potentially effective in CHF patients who require frequent administration of diuretic agents. However, there is no evidence clinical evidence on the clinical safety of SGLT2 inhibitors for heart failure, and it is possible excess intravascular fluid depletion may be exacerbated following administration of SGLT2 in patients with heart failure taking diuretics. Given this current status, it is necessary to assess the safety and efficacy of SGLT2 inhibitors for diabetic patients with CHF.

The results of the EMPA-REG OUTCOME trial showed that administration of empagliflozin significantly reduced CV adverse outcomes in T2DM patients with higher CV risk [13]. Compared to the placebo group, any cause of death including worsening of heart failure, was significantly lower in the empagliflozin group. In addition, a 35 % relative risk reduction in hospitalization for heart failure was observed in the empagliflozin group. Although a significant reduction in hospitalization for heart failure was clearly documented, participants with heart failure comprised only about 10 % of the trial population at baseline. Moreover, about 40 % of the participants were taking diuretics at baseline, and therefore how to use SGLT2 inhibitors in conjunction with diuretics was of great concern in the clinical management of these patients. Taken together, the therapeutic effects of SGLT2 inhibitors in specific patients with heart failure have yet to be established (Fig. 1). It is therefore necessary to assess the clinical safety and efficacy of SGLT2 inhibitors focusing on T2DM patients with CHF.

Among the SGLT family, SGLT2 plays a central role in glucose reuptake specifically in the proximal renal tubule with high-capacity and low-affinity. SGLT1 is also a glucose transporter with low-capacity and high-affinity and is expressed mainly in the intestine, kidney, and heart. Compared with other SGLT2 inhibitors, canagliflozin has a relatively lower selectivity for SGLT2 over SGLT1 [51], and as a result also inhibits SGLT1 in the upper intestine, causing a delayed postprandial hyperglycemic response, similar to that seen with alpha-glucosidase inhibitors [52]. In addition, intestinal SGLT1 inhibition enhances glucagon-like peptide-1 secretion from L-cells in the lower intestine where residual glucose may reach [53]. Based on these results, simultaneous inhibition of SGLT1 by canagliflozin may also contribute, in part, to treatment of T2DM, despite its possible gastrointestinal side effects such as diarrhea caused by glucose-galactose malabsorption. However, the potential impacts of SGLT1 inhibition by canagliflozin on heart tissue still remain uncertain, due to the high blood protein-binding of canagliflozin of about 99 %. Cardiac SGLT1 is highly expressed in myocyte salcolemma and significantly up-regulated according to the pathological condition of cardiomyocytes with altered glucose requirements that occurs in diabetic or ischemic cardiomyopathy and in failing hearts [54]. Because the expression level of cardiac SGLT1 correlates with cardiac pathogenesis, inhibitors of the SGLT family may exert favorable effects on cardiac diseases [55]. On the other hand, studies in mouse models of ischemia–reperfusion injury have shown cardiac SGLT1 may have a cardioprotective role by optimizing glucose uptake into heart tissue [56]. Further in vitro and in vivo studies are needed to determine the clinical effects of canagliflozin on cardiac SGLT1 inhibition.

There are several limitations in the trial. First, the CANDLE trial is not a placebo-controlled trial. The aim of the trial is therefore to test the safety and non-inferiority of canagliflozin compared with glimepiride in T2DM patients with CHF. Second, as the trial is an open label design, there may be some bias towards the assessment of outcomes resulting from the physicians’ choice of treatment. However, there are strict definitions on the background treatment that will, in principle and if possible, remain unchanged during the trial interval. To overcome this possible bias, NT-proBNP, a primary endpoint, will be measured at a central laboratory, and all the data will be managed and statistically analyzed in a blinded manner. Finally, the trial interval of 24 weeks may be short to titrate the appropriate dose of glimepiride. According to a previous study from Japan that evaluated the safety and efficacy of glimepiride titration using self-monitoring blood glucose (SMBG) method [57], glimepiride treatment during 24 weeks resulted in a sufficient reduction in HbA1c level in the SMBG group and even in patients in the control group who were titrated empirically by their clinician without causing severe hypoglycemia. The average doses of glimepiride were 1.0 ± 0.8 mg/day in the SMBG group and 0.6 ± 0.3 mg/day in the control group, with no patient receiving glimepiride ≥3.5 mg/day. In addition, the HbA1c level at 24 weeks was significantly lower in patients receiving 0.5 mg/day compared with patients receiving >0.5 mg/day. This result indicates that glimepiride treatment for 24 weeks may not necessarily be too short to titrate glimepiride. Inappropriate titration of glimepiride may impact glycemic control and NT-proBNP levels in the glimepiride group, although there is currently insufficient evidence, as to whether or not, or the extent to which glimepiride treatment may affect NT-proBNP levels.

In summary, accumulated evidence suggests that SGLT2 inhibitors may have considerable impact on clinical diabetes care, with the potential to markedly improve clinical outcomes in patients with T2DM, especially those at higher risk of CV events. It is therefore important to evaluate the clinical implication of SGLT2 inhibitors in T2DM patients with CHF. The CANDLE trial is the first to test the safety and non-inferiority of canagliflozin for T2DM patients with CHF. This study has the potential to provide novel clinical insights on the treatment of diabetic patients with high CV risk.

## Additional files

10.1186/s12933-016-0381-x  Manner of background and study drugs.
10.1186/s12933-016-0381-x  Guidepost for appropriate glycemic control in Japan.
10.1186/s12933-016-0381-x  Routine blood and urine examination.
10.1186/s12933-016-0381-x  Trial organization.

## Abbreviations

ANCOVA: analysis of covariance
ANGPTL2: angiopoietin-like protein 2
BP: blood pressure
BW: body weight
CEC: clinical events committee
CHF: chronic heart failure
CV: cardiovascular
DSMB: data and safety monitoring board
eGFR: estimated glomerular filtration rate
FAS: full analysis set
hsCRP: high-sensitivity C reactive protein
IL-8: interleukin-8
LVEF: left ventricular ejection fraction
MLHF: Minnesota living with heart failure
NT-proBNP: N-terminal pro-brain natriuretic peptide
NYHA: New York Heart Association
PTX3: pentraxin 3
RAGE: receptor for advanced glycation end products
SGLT1(or 2): sodium glucose co-transporter 1(or 2)
SMBG: self-monitoring blood glucose
T2DM: type 2 diabetes mellitus
