# Supplementary material for: Rationale and design of a randomized trial to test the safety and non-inferiority of canagliflozin in patients with diabetes with chronic heart failure: the CANDLE trial
Source: Cardiovasc Diabetol. 2016 Apr 4;15:57. doi: 10.1186/s12933-016-0381-x (PMC4820875; doi:10.1186/s12933-016-0381-x)
Supplement: Supplementary file 1 — 10.1186/s12933-016-0381-x Manner of background and study drugs. [file 12933_2016_381_MOESM1_ESM.pptx]

## Slide 1
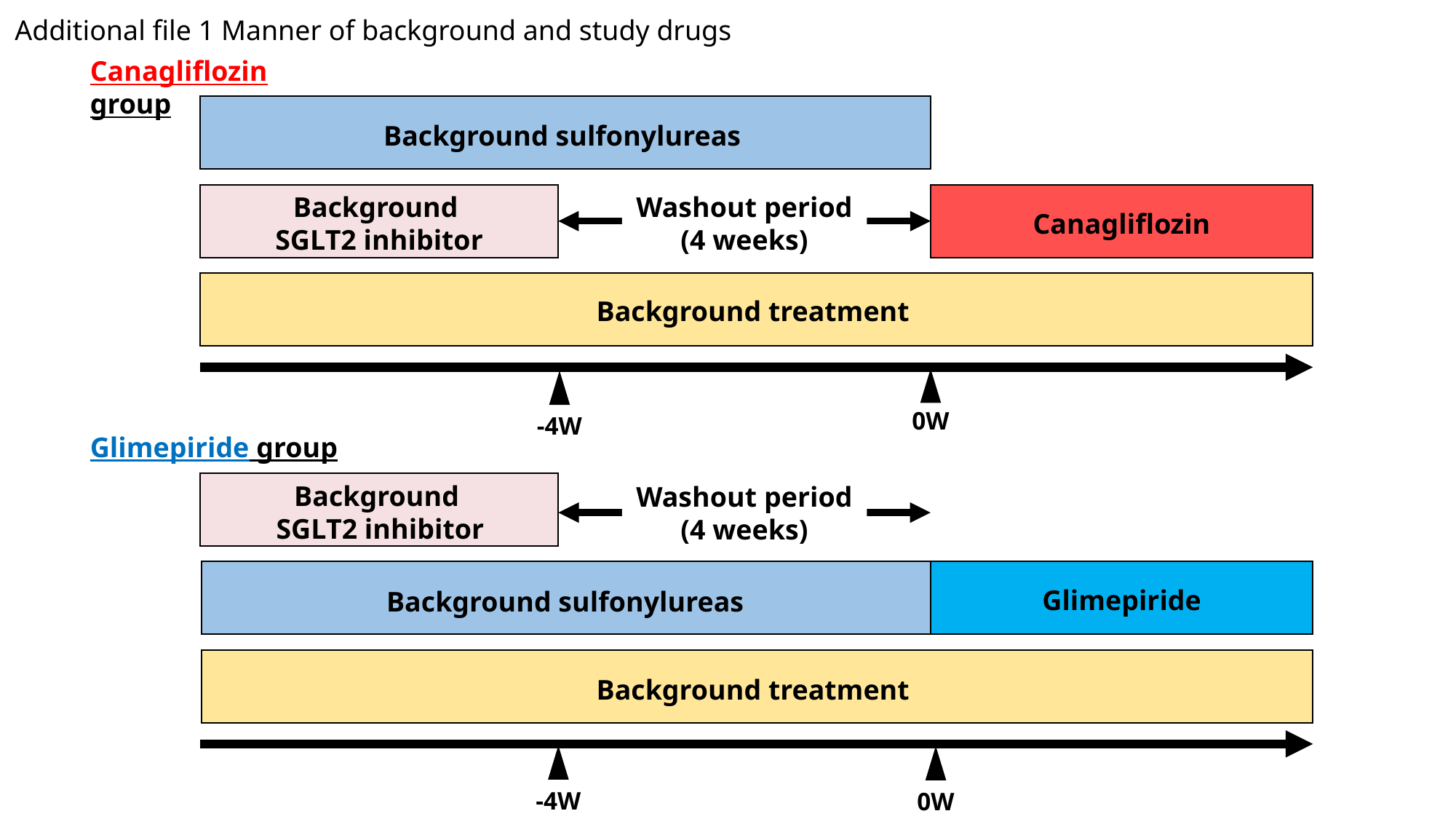

Additional file 1 Manner of background and study drugs
Canagliflozin group
Background sulfonylureas
Background
SGLT2 inhibitor
Washout period
(4 weeks)
Canagliflozin
Background treatment
-4W
0W
Glimepiride group
Background
SGLT2 inhibitor
Washout period
(4 weeks)
Glimepiride
Background sulfonylureas
Background treatment
-4W
0W
